# Age-Related Differences in Cardiovascular and Cerebrovascular Responses During the Head-Up Tilt Test: An Exploratory Study Using Continuous Biosignal Data

**DOI:** 10.3390/s24237565

**Published:** 2024-11-27

**Authors:** Catherine Park, Ji Man Hong

**Affiliations:** 1 Division of Digital Healthcare, Yonsei University, Wonju 26493, Republic of Korea; catherine.park@yonsei.ac.kr; 2 Department of Convergence of Healthcare and Medicine, Ajou University Graduate School of Medicine, Suwon 16499, Republic of Korea; 3 Department of Neurology, Ajou University School of Medicine, Suwon 16499, Republic of Korea

**Keywords:** head-up tilt, cardiovascular, cerebrovascular, age dependent response, time series analysis

## Abstract

The head-up tilt (HUT) test assesses both cardiovascular autonomic regulation and cerebral autoregulation. This exploratory study examined age-related changes in cardiovascular and cerebrovascular responses during the HUT test in three healthy cohorts (young, middle-aged, and elderly). We recruited 2342 neurologist-diagnosed ‘normal’ individuals from 18 June 2014 to 25 February 2022. Cerebrovascular and cardiovascular responses were assessed during the HUT test, including cerebral blood flow velocity (CBFv) of the middle cerebral artery, systolic arterial pressure (SYS), diastolic arterial pressure (DIA), mean arterial pressure (MAP), pulse pressure (PP), heart rate (HR), stroke volume (SV), cardiac output (CO), and cerebrovascular conductance (CVCi). These variables were analyzed across three groups (young, middle-aged, and elderly) and three periods (resting, post-HUT, and recovery). Participants were stratified into three age groups: young (18–45 years; *n* = 384), middle-aged (46–59 years; *n* = 434), and elderly (≥60 years; *n* = 590). PP increased significantly with age, while CBFv and CVCi decreased significantly across the three periods. As measurements progressed, DIA and HR increased, and SV, CBFv, and CVCi decreased. This study enhances our understanding of age-related differences in cardiovascular and cerebrovascular responses to the HUT test. These insights may improve the clinical utility of the HUT test and guide outcome analysis across age groups.

## 1. Introduction

Aging is associated with alterations in cardiovascular autonomic regulation and cerebral autoregulation. In the elderly, these changes can lead to decreased baroreflex sensitivity, altered balance between sympathetic and parasympathetic activity, impaired heart rate variability, and dysfunction in the autonomic neurotransmitter system [1,2,3,4]. Additionally, impaired cerebral autoregulation (the brain’s ability to maintain stable cerebral blood flow despite fluctuations in systemic blood pressure) can increase susceptibility to cerebral hypoperfusion, cerebrovascular events, and cognitive decline [5,6,7].

The head-up tilt (HUT) test, also known as the tilt table test, is a valuable tool for assessing both cardiovascular autonomic regulation and cerebral autoregulation. The test involves tilting the patient from a supine to an upright position, typically at an angle between 60 and 70 degrees [8,9,10]. This orthostatic stress causes a gravitational shift in blood volume, challenging the autonomic nervous system to maintain blood pressure and heart rate [11]. Consequently, the HUT test evaluates the integrity of autonomic responses and cerebral vessels’ ability to maintain perfusion under orthostatic stress [12]. In particular, the HUT test can evaluate the ability of cerebral vessels to constrict or dilate appropriately to maintain cerebral perfusion under orthostatic stress.

In clinical practice, the tilt table test has several benefits over measuring medical parameters while at rest (e.g., heart rate variability, baroreflex sensitivity, and cerebral blood flow velocity) for evaluating cardiovascular autonomic and cerebral autoregulation. The benefits include functional relevance [10], dynamic assessment [11], provocative testing [13], comprehensive evaluation (simultaneous assessment of multiple physiological parameters, including heart rate, blood pressure, symptoms, and cerebral perfusion) [11], diagnostic utility [14], and assessment of treatment response [15]. Consequently, the HUT test has been widely used to diagnose a variety of symptoms and conditions, primarily associated with autonomic nervous system dysfunction, including light-headedness, dizziness, orthostatic hypotension, vasovagal syncope, and postural orthostatic tachycardia syndrome [8,9,10,11,12,13,14,15].

Given the effectiveness of the HUT test and the substantial impact of aging on cardiovascular and cerebral functions [8,9,10,11,12,13,14,15,16,17], the aim of this exploratory study was to investigate the effects of age on cardiovascular and cerebrovascular responses during the HUT test. In particular, we measured and analyzed comprehensive physiological variables related to cardiovascular and cerebral function across three healthy cohorts (young, middle-aged, and elderly) to understand age-related differences.

This study provides a comprehensive analysis of cardiovascular and cerebrovascular responses during the HUT test across three age groups (young, middle-aged, and old) by identifying significant age-related differences in key physiological variables across three measurement periods (resting, post-HUT, and recovery periods). It also emphasizes within-group temporal changes and between-group differences. The findings of this study could potentially improve the clinical utility of the HUT test and serve as a reference for age-specific outcome analyses in both research and clinical settings.

The remainder of this paper is organized as follows: (1) Section 2 describes details on the materials and methods, including participant recruitment, ethical approval, experimental setup and protocols, and data and statistical analysis; (2) Section 3 reports the results of this study, including the demographic and clinical characteristics of the participants, as well as between-group and within-group comparisons of physiological variables across the measurement periods; (3) Section 4 discusses the main findings, describes the implications of the results, and addresses the limitations of this study; and (4) Section 5 concludes the paper by summarizing the key contributions of this study and suggesting directions for future research.

## 2. Materials and Methods

This section describes (1) the participants, including recruitment criteria, ethical approval, and procedural details for grouping participants; (2) the experimental apparatus, setup, and protocols with our methodological approach; (3) the variables and their definitions; and (4) the data and statistical analysis methods used for all variables.

### 2.1. Participants

This retrospective study analyzed electronic health records from Ajou University Hospital between 18 June 2014 and 25 February 2022. The study population was selected from patients who presented to the neurology outpatient department with complaints of dizziness and underwent a HUT test within two months of symptom onset. Among 2342 subjects who underwent the HUT test, 1408 were identified as normal according to the current guideline from the Consensus statement of the European Federation of Autonomic Societies (EFAS) endorsed by the American Autonomic Society (AAS) and the European Academy of Neurology (EAN). Exclusion criteria included (1) severe cardiogenic arrhythmia, epilepsy, encephalitis, or vertigo; (2) neurological diseases such as neurally mediated syncope, orthostatic hypotension, hyperventilation syndrome, postural orthostatic tachycardia syndrome; and (3) orthostatic dizziness. This retrospective study utilized electronic health records (EHRs) and was approved by the Institutional Review Board of Ajou University Medical Center (IRB#: AJOUIRB-DB-2023-529).

All participants were categorized into three groups (i.e., young, middle-aged, and old). The selected age thresholds (young: 18–45 years, middle-aged: 46–59 years, elderly: ≥60 years) align with previous studies that have documented distinct physiological stages associated with aging. Young adults (18–45 years) generally exhibit optimal autonomic function and cerebral perfusion [3], while middle-aged adults (46–59 years) show early signs of age-related declines in baroreflex sensitivity and cerebrovascular regulation [4]. The elderly group (≥60 years) was chosen based on evidence indicating significant decreases in cerebral blood flow and impaired autonomic responses, which are well documented in the geriatric population [4]. This categorization enables the examination of age-related differences in cardiovascular and cerebrovascular responses during the HUT test.

### 2.2. Experimental Apparatus and Procedure

Although bioimpedance-based sensors are increasingly being examined for non-invasive stroke volume and cardiac output measurement because they can estimate hemodynamic parameters from thoracic impedance changes due to the mechanical events of cardiac cycle [18,19], methods that measure bioimpedance are limited by their sensitivity to external factors such as changes in body position, respiratory movement, and variations in thoracic fluid content, which can reduce measurement accuracy, especially during dynamic tests like the HUT test [20]. However, based on these limitations, we used the Finometer^®^ Model-2, which is suitable for the volume-clamp method for continuous non-invasive measurement of stroke volume and cardiac output by using pulse contour analysis. This has been validated against invasive gold standards and is considered more accurate and reproducible with regard to beat-to-beat hemodynamic fluctuations, especially during orthostatic challenges [21,22]. The Finometer^®^ Model-2 (FMS Medical System, Amsterdam, The Netherlands) continuously measures arterial pressure. This is especially important during a HUT test where rapid physiological responses need to be evaluated. Additionally, the chosen digital transcranial doppler system (ST3 Digital Transcranial Doppler System, Spencer Technologies, Seattle, WA, USA) was designed for continuous monitoring of cerebral blood flow velocity, making it a suitable companion to enhance the cardiovascular measurements with a complete assessment of concurrent cardiovascular and cerebrovascular responses [23].

All participants were placed in this supine posture while baseline cardiovascular and cerebrovascular responses were assessed using the Finometer^®^ Model-2 and the ST3 Digital Transcranial Doppler System, respectively. Subsequently, all participants underwent passive tilting to approximate an upright posture (70° degrees) in order to replicate standing for a duration of 15 min. After the tilt phase for 15 min, the table is returned to the horizontal position.

### 2.3. Variables

**Figure 1:**
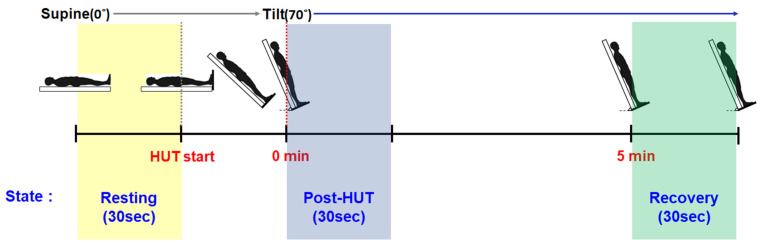
Description of measurement periods (i.e., resting (30 s before the HUT), post-HUT (30 s after the HUT), and recovery (30 s after 5-min post-HUT)).

Using the ST3 Digital Transcranial Doppler System, we measured cerebrovascular responses such as cerebral blood flow velocity (CBFv). We also measured cardiovascular responses such as systolic arterial pressure (SYS), diastolic arterial pressure (DIA), mean arterial pressure (MAP), pulse pressure (PP), heart rate (HR), stroke volume (SV), and cardiac output (CO) assessed by the Finometer^®^ Model-2. Furthermore, we computed the cerebrovascular conductance (CVCi) by dividing CBFv by MAP. Table 1 provides the description and definition of the 9 variables used to assess cardiovascular and cerebrovascular responses. Figure 1 presents the measurement periods, namely, resting (30 s before the HUT), post-HUT (30 s after the HUT), and recovery (30 s after 5-min post-HUT) during the HUT test.

### 2.4. Data and Statistical Analysis

MATLAB 2023b (MathWorks, Natick, MA, USA) and IBM SPSS Statistics 27 (IBM Corp., Armonk, NY, USA) were used to conduct data and statistical analysis. Outcome measures were the participants’ demographics (i.e., age, sec, and BMI) and the 9 variables (i.e., SYS, DIA, MAP, PP, HR, SV, CO, CBFv, and CVCi) computed for the 3 periods (i.e., resting, post-HUT, and recovery).

We used the Shapiro–Wilk test to determine the normal distribution of continuous variables. We conducted a one-way analysis of variance (ANOVA) for normally distributed variables or a Mann–Whitney U test for non-normally distributed variables to determine mean differences between groups (i.e., young, middle-aged, and old). We performed a chi-square test to test categorical variables for significant levels between groups. By adjusting for sex and BMI as potential confounders, we used a linear mixed model (LMM) for normally distributed variables and generalized estimating equations (GEEs) for non-normally distributed variables to assess the main effects of the measurement period (i.e., resting, post-HUT, and recovery) and the group (young, middle-aged, and old), because the linear mixed model and generalized estimating equations allowed for testing differences between groups, as well as at specific time points for the longitudinal design, and were valid for missing data for a particular patient or time at random. To assess the change in cardiovascular and cerebrovascular responses during the HUT test, we compared the nine cardiovascular and cerebrovascular variables for the three measurement periods for three age-dependent groups. For this analysis, we conducted multiple pairwise comparisons using the least significant difference method. The significance level was set at 2-sided *p* < 0.05 for all statistical analyses.

## 3. Results

This section presents (1) the demographic and clinical characteristics of the study participants; and (2) between-group and within-group comparisons of all variables across the three measurement periods (i.e., resting, post-HUT, and recovery periods).

### 3.1. Demographic and Clinical Characteristics

Out of 1408 included participants, 384 (27.3%) were young, 434 (30.8%) were middle-aged, and 590 (41.9%) were old. Table 2 presents demographic and clinical characteristics with corresponding statistical results, showing significant differences between the groups.

### 3.2. Between-Group Comparison

**Figure 2:**
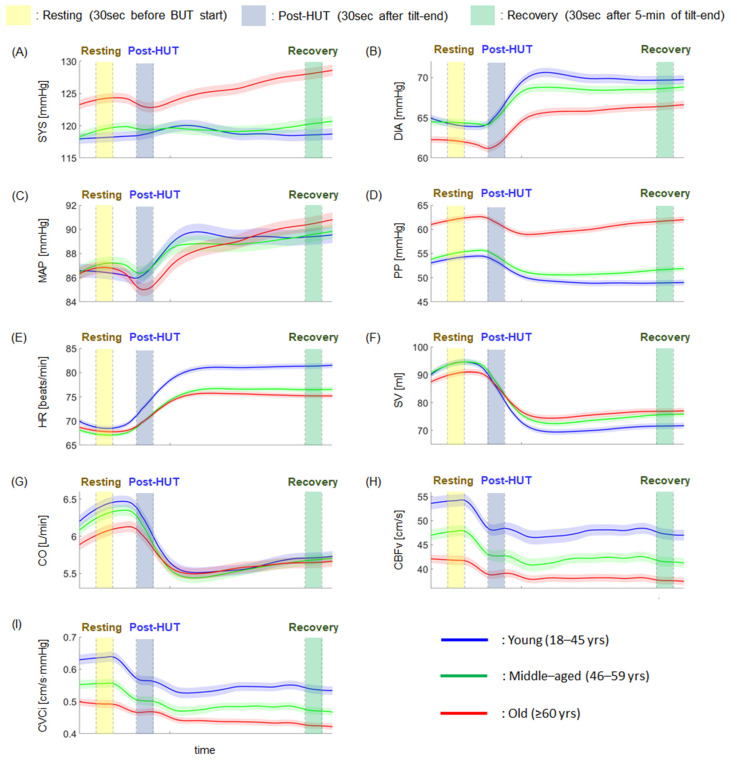
Results of each variable as a function of time during the HUT test for three groups. (**A**) SYS (systolic arterial pressure). (**B**) DIA (diastolic arterial pressure). (**C**) MAP (mean arterial pressure). (**D**) PP (pulse pressure). (**E**) HR (heart rate). (**F**) SV (stroke volume). (**G**) CO (cardiac output). (**H**) CBFv (cerebral blood flow velocity). (**I**) CVCi (cerebrovascular conductance).

Table 2 and Figure 2 report the results of statistical analysis for nine variables between three groups for three measurement periods. Across the three periods, PP was significantly higher with age, while CBFv and CVCI were significantly lower.

Except for a comparison between the young and middle-aged groups during the post-HUT period, SYS was significantly higher with age in all periods. DIA was significantly lower as age increased, whereas there was no statistical difference between the young and middle-aged groups during the resting and recovery periods.

MAP was significantly higher for the middle-aged group compared to the young and old groups during the resting period, and was significantly lower for the old groups during the post-HUT period compared to the young and middle-aged groups. However, MAP was not significantly different between the young and old groups during the resting period, between the young and middle-aged groups during the post-HUT period, and among the three groups during the recovery period.

Except for the comparison between the middle-aged and old groups during the resting and post-HUT periods, HR was significantly lower as age increased. SV was significantly higher with age during the post-HUT and recovery periods. However, SV was not significantly different among the three groups during the resting period. CO was significantly higher for the young group compared to the middle-aged and old groups during the resting period. Additionally, the young group showed a significantly higher CO compared to the middle-aged group during the recovery period.

### 3.3. Within-Group Comparison

Table 3 and Figure 2 report the results of statistical analysis for nine variables between three measurement periods for three groups. Among the nine variables, DIA, HR, SV, CBFv, and CVCi were significantly different between three measurement periods in the three groups. As the measurement period proceeded, DIA and HR, in particular, were significantly increased, and SV, CBFv, and CVCi were significantly decreased.

In the young group, SYS was significantly increased during the post-HUT period compared to the resting and recovery periods. However, it was not statistically different between the resting and recovery periods. As the measurement period proceeded, MAP was significantly increased and PP was significantly decreased. CO was significantly decreased during the post-HUT period compared to the resting and recovery periods. Additionally, the recovery period showed a significant decrease in CO compared to the resting period.

In the middle-aged group, the recovery period showed a significant decrease in SYS compared to the post-HUT period. However, it was not statistically different between the resting and post-HUT periods and the resting and recovery periods. MAP was significantly increased as the measurement period proceeded. PP was significantly decreased during the post-HUT and recovery periods compared to the resting period. However, it was not statistically different between the post-HUT and recovery periods. Furthermore, CO was significantly decreased during the post-HUT period compared to the resting and recovery periods and during the recovery period compared to the resting period.

In the old group, SYS was significantly decreased during the post-HUT period compared to the resting and recovery periods. Additionally, the recovery period showed a significant decrease in SYS compared to the resting period. MAP was significantly decreased during the post-HUT period compared to the resting and recovery periods, and was significantly increased during the recovery period compared to the resting period. PP was significantly decreased during the post-HUT period compared to the resting and recovery periods. However, it was not significantly different between the resting and recovery periods.

## 4. Discussion

This study quantitatively assessed cerebrovascular and cardiovascular responses during the HUT test across three large healthy cohorts. We measured, analyzed, and compared nine variables (i.e., SYS, DIA, MAP, PP, HR, SV, CO, CBFv, and CVCi) across three measurement periods (i.e., resting, post-HUT, and recovery) and three groups (i.e., young, middle-aged, and old). The results of this study revealed significant effects of age and measurement period on cerebrovascular and cerebrovascular responses during the HUT test.

The main results showed that CBFv and CVCi were significantly lower, while PP was significantly higher during all measurement periods as age increased. Additionally, SYS during the resting and recovery periods and SV during the post-HUT and recovery periods were significantly higher with age. Conversely, DIA during the post-HUT period and HR during the recovery period were significantly lower in older groups. These changes can be attributed to age-related vascular and cardiac function alterations, aligning with previous studies [24,25,26]. Indeed, age-related changes may impair cerebral autoregulation and less-efficient adaptation to postural changes.

Furthermore, across all age groups, CBFv, CVCi, and SV significantly decreased, while DIA and HR increased as the measurement period proceeded. The decrease in CBFv and CVCi during the HUT test supports previous findings that cerebral autoregulation is compromised with increased verticalization [26]. The observed reduction in stroke volume is consistent with the gravitational shift of blood volume away from the heart and central circulation during the HUT test [27]. The increase in DIA and HR can be explained by autonomic responses aimed at maintaining blood pressure and flow despite blood pooling in the lower extremities [28,29]. These findings confirm that postural changes significantly affect cerebrovascular and cardiovascular dynamics, regardless of age.

This study has several limitations. First, as a retrospective study, it relies on the accuracy and completeness of electronic health records. Second, the exclusion criteria, while necessary to identify normal subjects, may limit the generalizability of the findings to broader populations. Third, the study did not account for potential confounding factors such as medication use, lifestyle differences, or comorbidities that could influence cardiovascular and cerebrovascular responses. Fourth, gender differences and baseline blood pressure effects, known to impact autonomic and hemodynamic responses, were not specifically analyzed in this study. Fifth, the exclusion of individuals with conditions like orthostatic dizziness, epilepsy, cardiac arrhythmias, and mild autonomic or cerebrovascular dysfunction may further limit the applicability of the results to clinical populations. Lastly, the reliance on three discrete time points (resting, post-HUT, and recovery) may have overlooked dynamic changes occurring outside these specific periods, potentially missing subtle temporal dynamics in physiological responses.

Despite these limitations, the findings of this study provide important baseline data associated with age-related cardiovascular and cerebrovascular responses in a neurologically normal population. Studies need to overcome these limitations to improve the transferability of results clinically. Primarily, a prospective study design would provide better control over confounding and allow for causal inferences about age differences. Broadening the inclusion criteria to include those with mild autonomic or cerebrovascular dysfunction would create a more representative clinical sample and increase the generalizability of findings. Further studies exploring gender-specific analyses and baseline blood pressure measurements may help to clarify their impact on autonomic and hemodynamic responses. Additionally, measuring data on potential confounders (i.e., history of medication use, physical activity, and hydration status) would allow for a deeper understanding of the effects seen. Moreover, a continuous time-series analysis over the duration of the HUT test might identify dynamic responses and potentially provide greater granularity regarding cardiovascular and cerebrovascular response function. Lastly, the integration of adjunct markers such as baroreflex sensitivity and autonomic nervous system biomarkers would provide insight into potential physiological mechanisms that may mediate age-related differences. Taken together, these approaches would enrich knowledge on developmental differences and increase generalizability across healthy populations, as well as clinical samples.

## 5. Conclusions

This exploratory study investigated cardiovascular and cerebrovascular responses during the HUT test across different age groups and measurement periods. Significant changes in CBFv, CVCi, and PP were observed with increasing age, while CBFv, CVCi, SV, DIA, and HR varied significantly across measurement periods in all groups. These findings suggest that these variables could be crucial in assessing cardiovascular and cerebrovascular responses during the HUT test. Our results enhance the understanding of age-related differences in these responses and support the clinical utility of the HUT test in diagnosing and managing conditions related to autonomic dysfunction. Future research should consider longitudinal studies to explore causal relationships and the impact of potential confounders on these responses.

## Figures and Tables

**Table 1:** Description of cardiovascular and cerebral blood flow variables.

Name	Unit	Description
Systolic arterial pressure (SYS)	mmHg	Maximum pressure in arterial systole
Diastolic arterial pressure (DIA)	mmHg	Lowest pressure just before the next upstroke
Mean arterial pressure (MAP)	mmHg	Average pressure between upstrokes
Pulse pressure (PP)	mmHg	Differences between systolic pressure and diastolic pressure PP = Systolic arterial pressure (SYS) − Diastolic arterial pressure (DIA)
Heart rate (HR)	beats/min	Pulse rate derived from inter-beat interval
Stroke volume (SV)	mL	Amount of blood ejected from the ventricle with each cardiac cycle SV = Cardiac Output (CO)/Heart rate (HR)
Cardiac output (CO)	L/min	Product of stroke volume and heart rate CO = Stroke volume (SV) × Heart rate (HR)
Cerebral blood flow velocity (CBFv)	cm/s	Bilateral middle cerebral artery blood flow velocities the rate of delivery of arterial blood to a capillary bed in tissue
CVCi	cm/s∙mmHg	Cerebrovascular conductance calculated by dividing CBFv by mean arterial pressure (MAP)

**Table 2:** Statistical results of nine variables between three groups (i.e., young, middle-aged, and old) for three measurement periods (i.e., resting, post-HUT, and recovery). Values are presented as mean ± standard error. An asterisk denotes statistical significance.

	Resting			*p* Value			Post-HUT			*p* Value			Recovery			*p* Value		
Variable	Young	Middle	Old	Young vs. Middle	Young vs. Old	Middle vs. Old	Young	Middle	Old	Young vs. Middle	Young vs. Old	Middle vs. Old	Young	Middle	Old	Young vs. Middle	Young vs. Old	Middle vs. Old
SYS	116.6 ± 0.7	119.7 ± 0.7	125.4 ± 0.8	0.001 *	<0.0001 *	<0.0001 *	119.0 ± 0.8	119.4 ± 0.9	123.6 ± 0.9	0.711	<0.0001 *	<0.0001 *	116.7 ± 0.8	120.6 ± 0.8	131.2 ± 0.9	0.001 *	<0.0001 *	<0.0001 *
DIA	64.3 ± 0.4	65.0 ± 0.4	61.0 ± 0.4	0.306	<0.0001 *	<0.0001 *	70.2 ± 0.5	68.3 ± 0.5	62.3 ± 0.5	0.013 *	<0.0001 *	<0.0001 *	70.8 ± 0.5	69.7 ± 0.5	66.1 ± 0.5	0.125	<0.0001 *	<0.0001 *
MAP	85.9 ± 0.5	87.7 ± 0.5	86.2 ± 0.5	0.016 *	0.641	0.049 *	88.9 ± 0.6	88.5 ± 0.6	85.2 ± 0.6	0.652	<0.0001 *	<0.0001 *	89.7 ± 0.6	90.5 ± 0.6	91.2 ± 0.6	0.325	0.080	0.431
PP	52.3 ± 0.4	54.8 ± 0.5	64.5 ± 0.6	<0.0001 *	<0.0001 *	<0.0001 *	48.8 ± 0.4	51.1 ± 0.6	61.4 ± 0.6	0.001 *	<0.0001 *	<0.0001 *	45.9 ± 0.4	50.9 ± 0.5	65.1 ± 0.7	<0.0001 *	<0.0001 *	<0.0001 *
HR	69.8 ± 0.5	67.3 ± 0.5	67.7 ± 0.5	<0.0001 *	0.003 *	0.520	79.1 ± 0.6	73.3 ± 0.5	72.4 ± 0.5	<0.0001 *	<0.0001 *	0.192	83.8 ± 0.6	76.3 ± 0.5	74.3 ± 0.5	<0.0001 *	<0.0001 *	0.006 *
SV	93.7 ± 1.0	93.6 ± 1.0	91.1 ± 1.2	0.925	0.082	0.100	73.0 ± 0.9	76.7 ± 1.0	80.4 ± 1.2	0.005 *	<0.0001 *	0.014 *	70.5 ± 0.9	74.7 ± 0.9	78.0 ± 1.2	0.001 *	<0.0001 *	0.024 *
CO	6.5 ± 0.1	6.3 ± 0.1	6.1 ± 0.1	0.025 *	<0.0001 *	0.058	5.7 ± 0.1	5.5 ± 0.1	5.7 ± 0.1	0.150	0.889	0.159	5.8 ± 0.1	5.6 ± 0.1	5.7 ± 0.1	0.038 *	0.138	0.681
CBFv	56.9 ± 1.3	47.4 ± 1.1	40.3 ± 0.9	<0.0001 *	<0.0001 *	<0.0001 *	51.8 ± 1.2	43.0 ± 1.1	37.9 ± 0.8	<0.0001 *	<0.0001 *	<0.0001 *	50.0 ± 1.1	41.4 ± 1.0	36.7 ± 0.7	<0.0001 *	<0.0001 *	<0.0001 *
CVCi	0.672 ± 0.016	0.547 ± 0.013	0.479 ± 0.012	<0.0001 *	<0.0001 *	<0.0001 *	0.591 ± 0.014	0.494 ± 0.013	0.458 ± 0.010	<0.0001 *	<0.0001 *	0.027 *	0.566 ± 0.013	0.465 ± 0.012	0.414 ± 0.009	<0.0001 *	<0.0001 *	<0.0001 *

Note: SYS: systolic arterial pressure; DIA: diastolic arterial pressure; MAP: mean arterial pressure; PP: pulse pressure; HR: heart rate; SV: stroke volume; CO: cardiac output; CBFv: cerebral blood flow velocity; CVCi: cerebrovascular conductance.

**Table 3:** Statistical results of nine variables between three measurement periods (i.e., resting, post-HUT, and recovery) for three groups (i.e., young, middle-aged, and old). Values are presented as mean ± standard error. An asterisk denotes statistical significance.

	Young Group (*n* = 384)			*p* Value		
Variable	Resting	Post-HUT	Recovery	Resting vs. Post-HUT	Resting vs. Recovery	Post-HUT vs. Recovery
SYS	116.6 ± 0.7	119.0 ± 0.8	116.7 ± 0.8	<0.0001 *	0.898	<0.0001 *
DIA	64.3 ± 0.4	70.2 ± 0.5	70.8 ± 0.5	<0.0001 *	<0.0001 *	0.015 *
MAP	85.9 ± 0.5	88.9 ± 0.6	89.7 ± 0.6	<0.0001 *	<0.0001 *	0.014 *
PP	52.3 ± 0.4	48.8 ± 0.4	45.9 ± 0.4	<0.0001 *	<0.0001 *	<0.0001 *
HR	69.8 ± 0.5	79.1 ± 0.6	83.8 ± 0.6	<0.0001 *	<0.0001 *	<0.0001 *
SV	93.7 ± 1.0	73.0 ± 0.9	70.5 ± 0.9	<0.0001 *	<0.0001 *	<0.0001 *
CO	6.5 ± 0.1	5.7 ± 0.1	5.8 ± 0.1	<0.0001 *	<0.0001 *	<0.0001 *
CBFv	56.9 ± 1.3	51.8 ± 1.2	50.0 ± 1.1	<0.0001 *	<0.0001 *	<0.0001 *
CVCi	0.672 ± 0.016	0.591 ± 0.014	0.566 ± 0.013	<0.0001 *	<0.0001 *	<0.0001 *
	Middle-aged group (*n* = 434)					
	Resting	Post-HUT	Recovery	Resting vs. Post-HUT	Resting vs. Recovery	Post-HUT vs. Recovery
SYS	119.7 ± 0.7	119.4 ± 0.9	120.6 ± 0.8	0.470	0.087	0.005 *
DIA	65.0 ± 0.4	68.3 ± 0.5	69.7 ± 0.5	<0.0001 *	<0.0001 *	<0.0001 *
MAP	87.7 ± 0.5	88.5 ± 0.6	90.5 ± 0.6	0.021 *	<0.0001 *	<0.0001 *
PP	54.8 ± 0.5	51.1 ± 0.6	50.9 ± 0.5	<0.0001 *	<0.0001 *	0.600
HR	67.3 ± 0.5	73.3 ± 0.5	76.3 ± 0.5	<0.0001 *	<0.0001 *	<0.0001 *
SV	93.6 ± 1.0	76.7 ± 1.0	74.7 ± 0.9	<0.0001 *	<0.0001 *	<0.0001 *
CO	6.3 ± 0.1	5.5 ± 0.1	5.6 ± 0.1	<0.0001 *	<0.0001 *	<0.0001 *
CBFv	47.4 ± 1.1	43.0 ± 1.1	41.4 ± 1.0	<0.0001 *	<0.0001 *	<0.0001 *
CVCi	0.547 ± 0.013	0.494 ± 0.013	0.465 ± 0.012	<0.0001 *	<0.0001 *	<0.0001 *
	Old group (*n* = 590)					
	Resting	Post-HUT	Recovery	Resting vs. Post-HUT	Resting vs. Recovery	Post-HUT vs. Recovery
SYS	125.4 ± 0.8	123.6 ± 0.9	131.2 ± 0.9	<0.0001 *	<0.0001 *	<0.0001 *
DIA	61.0 ± 0.4	62.3 ± 0.5	66.1 ± 0.5	<0.0001 *	<0.0001 *	<0.0001 *
MAP	86.2 ± 0.5	85.2 ± 0.6	91.2 ± 0.6	0.003 *	<0.0001 *	<0.0001 *
PP	64.5 ± 0.6	61.4 ± 0.6	65.1 ± 0.7	<0.0001 *	0.107	<0.0001 *
HR	67.7 ± 0.5	72.4 ± 0.5	74.3 ± 0.5	<0.0001 *	<0.0001 *	<0.0001 *
SV	91.1 ± 1.2	80.4 ± 1.2	78.0 ± 1.2	<0.0001 *	<0.0001 *	<0.0001 *
CO	6.1 ± 0.1	5.7 ± 0.1	5.7 ± 0.1	<0.0001 *	<0.0001 *	0.619
CBFv	40.3 ± 0.9	37.9 ± 0.8	36.7 ± 0.7	<0.0001 *	<0.0001 *	<0.0001 *
CVCi	0.479 ± 0.012	0.458 ± 0.010	0.414 ± 0.009	<0.0001 *	<0.0001 *	<0.0001 *

Note: SYS: systolic arterial pressure; DIA: diastolic arterial pressure; MAP: mean arterial pressure; PP: pulse pressure; HR: heart rate; SV: stroke volume; CO: cardiac output; CBFv: cerebral blood flow velocity; CVCi: cerebrovascular conductance.

## Data Availability

Data cannot be shared publicly due to regulations from the Institutional Review Boards of Ajou University Medical Center (contact via phone: +82-31-219-7626, email: ajouirb@aumc.ac.kr). This is because the data contain potentially identifying or sensitive patient information, and distributing these data could breach patient confidentiality. Data are available from the aforementioned Institutional Review Boards for researchers who meet the criteria for access to confidential data.
